# FGFR1/YAP1 signaling in endothelial cells drives renal fibrosis and offers a therapeutic target

**DOI:** 10.3389/fphar.2026.1766265

**Published:** 2026-04-13

**Authors:** Xin Jiang, Yafeng Ren, Mengli Yan, Mingjing Li, Huixia Cao, Fengmin Shao

**Affiliations:** 1 Department of Nephrology, Zhengzhou University People’s Hospital, Henan Provincial People’s Hospital, Zhengzhou, China; 2 Henan Provincial Key Laboratory of Kidney Disease Innovation and Translation, Henan International Joint Laboratory of Kidney Disease and Microenvironment, Henan Provincial Clinical Research Center for Kidney Disease, Henan Provincial People’s Hospital, Zhengzhou, China; 3 Department of Nephrology, Xinxiang Medical University, Henan Provincial People’s Hospital, Zhengzhou, China

**Keywords:** chronic kidney disease (CKD), endothelial cells, fibroblast growth factorreceptor 1 (FGFR1), renal fibrosis, therapeutictarget, transforming growth factor-β (TGF-β), yes-associated protein 1(YAP1)

## Abstract

Renal fibrosis is the common pathological pathway for all chronic kidney diseases (CKD) progressing to end-stage renal failure, yet no current therapies can directly halt or reverse this process. Yes-associated protein 1 (YAP1), a core effector of the Hippo pathway, is an established driver of fibrosis, yet it presents a formidable challenge for direct pharmacological inhibition due to its structural and functional properties. This study aims to investigate the role of fibroblast growth factor receptor 1 (FGFR1) as an upstream regulator of YAP1 in renal fibrosis and to evaluate the therapeutic potential of targeting this signaling axis. We analyzed human fibrotic kidney samples, a unilateral ureteral obstruction (UUO) mouse model, and in vitro human umbilical vein endothelial cells (HUVECs), combined with genetic, pharmacological, and biochemical techniques, including endothelial-specific gene knockout, inhibitor assays, immunofluorescence, Western blot, and quantitative real-time PCR (qPCR). We found that FGFR1 and YAP1 were coordinately upregulated in the endothelial cells of fibrotic kidneys. Mechanistically, transforming growth factor-β (TGF-β) activated the FGFR1-ERK-YAP1 signaling cascade, which drove endothelial-to-mesenchymal transition (EndMT), inflammatory responses, and endothelial dysfunction. In vitro, both pharmacological inhibition of FGFR1 with PD173074 and genetic knockdown of FGFR1 or YAP1 effectively blocked this pro-fibrotic cascade. Consistent with in vitro findings, in the UUO mouse model, endothelial-specific deletion of YAP1 or administration of PD173074 significantly attenuated renal fibrosis, inflammatory responses, and vascular dysfunction, while preserving renal function. In addition, the pro-fibrotic function of this axis was further validated in a diabetic kidney disease mouse model. In conclusion, this study identifies the endothelial FGFR1/YAP1 axis as one of the important pro-fibrotic drivers in renal fibrosis progression and proposes an innovative therapeutic concept: indirectly modulating the “undruggable” transcriptional co-activator YAP1 by targeting its upstream, pharmacologically tractable receptor FGFR1. This strategy provides a novel interventional approach and potential target for anti-fibrotic intervention in CKD.

## 1 Introduction

Chronic kidney disease (CKD) afflicts approximately 13% of the global population, and its incidence continues to rise due to population aging and the increasing prevalence of comorbidities such as obesity, diabetes, and hypertension, imposing an increasingly severe burden (Kovesdy, 2025; Rao et al., 2025; Tangri et al., 2025) and emerging as one of the most challenging public health issues worldwide (Francis et al., 2024). China, as one of the countries with a heavy burden of CKD, has a prevalence of 8.2%, with over 80 million patients. However, awareness among adults is only 10%, and about 26.8% of patients have already progressed to advanced stages (Tangri et al., 2025; Francis et al., 2024; Wang et al., 2023; Wang et al., 2023). This profile of ‘high prevalence, low awareness, and low diagnosis and treatment rates’, compounded by the asymptomatic nature of early-stage disease, highlights the imperative not only to strengthen early prevention and screening systems but also to focus on the approximately one-third of patients in advanced stages. Renal fibrosis is the common and ultimate pathway in the progression of CKD, regardless of etiology, and represents the core pathological change leading to end-stage renal disease (ESRD) (Peng et al., 2023). In early CKD, kidney injury triggers extracellular matrix (ECM) accumulation. While this limited fibrotic response may serve a compensatory reparative function in the short term, prolonged, unregulated ECM deposition leads to irreversible architectural disruption and decline in renal function, culminating in kidney failure (Nastase et al., 2018; Humphreys and Julius, 2018). Current clinical management primarily focuses on controlling underlying conditions such as hyperglycemia and hypertension, which can only slow the progression of fibrosis; no therapeutics are available that can directly block or reverse this process (Abbad et al., 2025). Therefore, developing effective anti-fibrotic therapies is critical for halting the progression of CKD to uremia/ESRD.

The characteristic pathological features of renal fibrosis include tubular atrophy, chronic interstitial inflammation and fibrosis, glomerulosclerosis, and vascular rarefaction (Huang et al., 2023). However, due to the limited number of glomeruli obtainable from biopsy specimens and the relatively low abundance of endothelial cells in the renal parenchyma, the mechanisms underlying glomerulosclerosis and intrarenal vascular rarefaction remain less understood than those of tubulointerstitial fibrosis. This knowledge gap underscores the need to elucidate these under-investigated aspects of renal pathology. Glomerular and peritubular capillaries are essential for maintaining normal renal architecture and function. Injury to their endothelial cells and the consequent capillary loss are recognized as pivotal events that accelerate the progression of both glomerulosclerosis and tubulointerstitial fibrosis across various kidney diseases (Steegh et al., 2024; Bábíčková et al., 2017). This discovery revealed a direct mechanism by which endothelial cells participate in fibrogenesis. In 2008, Zeisberg et al. provided the first evidence that endothelial cells contribute to renal fibrosis via endothelial-mesenchymal transition (EndMT) across multiple murine models, including unilateral ureteral obstruction (UUO), streptozotocin (STZ)-induced diabetic nephropathy, and Alport nephropathy (Zeisberg et al., 2008). Subsequent research solidified the role of endothelial dysfunction as an early event in CKD. For instance, studies by Kristensen et al. indicated that in response to injuries such as hypertension and hyperglycemia, endothelial cells express adhesion molecules like VCAM-1, recruiting inflammatory cells to renal tissue. This process initiates and amplifies a chronic inflammatory response, thereby driving fibrosis (Kristensen et al., 2025). Beyond EndMT and inflammatory recruitment, an imbalance between pro-angiogenic and anti-angiogenic factors also contributes to CKD progression by disrupting endothelial cell proliferation and apoptosis (Tanabe et al., 2020). Therefore, endothelial cells function not merely as passive targets of chronic injury but as pivotal active contributors that integrate systemic and local damage signals to propel CKD progression. However, the precise molecular switches that convert ECs from quiescent linings into pro-fibrotic effectors are incompletely understood.

The mammalian Hippo pathway is a conserved serine/threonine kinase cascade. At its core, the MST-LATS kinase axis phosphorylates and thereby inhibits the transcriptional coactivators YAP1 and TAZ (Zhao et al., 2011; Ma et al., 2019). Functioning as a central growth-regulatory network, this pathway integrates diverse extracellular and intracellular cues to govern pivotal processes including organ size control, tissue regeneration, mechanosensing, and tumor suppression (Ma et al., 2019; Guo et al., 2025; Dupont et al., 2011). In the context of fibrosis, YAP1 has emerged as a central profibotic mediator. It not only engages in intricate crosstalk with the canonical TGF-β/Smad signaling axis to drive extracellular matrix (ECM) protein synthesis (Szeto et al., 2016; Patel et al., 2019; Feng et al., 2018), but also modulates mechanotransduction, tissue remodeling, and cellular energy stress responses (Szeto et al., 2016; Piccolo et al., 2014; Preisser et al., 2016; Liang et al., 2017). Consequently, YAP1 is widely recognized as a critical driver in the pathogenesis of renal fibrosis (Noguchi et al., 2018; Li et al., 2023; Xia et al., 2025; Wang et al., 2026). However, we also note that the functional role of YAP1 is highly context-dependent, exhibiting remarkable complexity and even opposing effects across different cell types and disease settings. For instance, in pulmonary fibrosis, YAP1 has been reported to mitigate fibrogenesis by suppressing alveolar epithelial cell senescence in one study (Su et al., 2024), yet another study demonstrates that YAP1 activation in fibroblasts exacerbates the fibrotic response (Liu et al., 2015). This functional heterogeneity underscores the likelihood that cell type-specific regulatory mechanisms are pivotal in dictating YAP1’s ultimate pathological output. In the context of renal fibrosis, the precise role of endothelial YAP1 remains elusive, and it is still unclear whether its inhibition confers renoprotection or aggravates disease progression. Additionally, targeting YAP1 therapeutically also poses substantial challenges. These stem from YAP1’s position at the nexus of multiple signaling pathways and its lack of a druggable active pocket (Awoonor-Williams et al., 2023; Yu et al., 2025); consequently, no highly specific YAP1 inhibitors have been successfully translated to the clinic. Verteporfin, a clinical agent used in ophthalmologic photodynamic therapy, is frequently employed as a experimental YAP1 inhibitor in renal fibrosis research (Szeto et al., 2016; Jin et al., 2020; Chen and Harris, 2016; Chen et al., 2020). However, its utility is questionable. Qi et al. reported that in diabetic nephropathy, verteporfin influenced tubular dedifferentiation via the TGF-β1/Smad/LATS1 axis but independently of the YAP1-TEAD-CTGF pathway; while it lowered serum creatinine in diabetic mice, it failed to ameliorate proteinuria or prevent podocyte loss (Qi et al., 2022). Corroborating this, Zhuang et al. demonstrated that YAP1 inhibition by verteporfin exacerbated podocyte apoptosis (Zhuang et al., 2021). These findings underscore a fundamental challenge: given the central role of Hippo/YAP1 in cellular homeostasis, systemic inhibition is likely to elicit widespread off-target effects. Therefore, comprehensive YAP1 blockade is an impractical strategy, and a cell-type-specific targeting approach may offer a more viable therapeutic window. In this regard, the work by Huang et al., which alleviated endothelial inflammation by specifically targeting endothelial YAP1/TAZ, provides a compelling precedent and a strategic direction (Huang et al., 2024). Building on this paradigm, we aim to investigate the role of endothelial YAP1 in renal fibrosis and explore its potential as a cell-selective therapeutic target.

Fibroblast growth factor receptor 1 (FGFR1), a transmembrane receptor tyrosine kinase, regulates critical cellular processes including proliferation, survival, and migration (Turner and Grose, 2010). In the context of fibrotic diseases, endothelial FGFR1 has been implicated in pathological angiogenesis that promotes liver fibrosis (Ding et al., 2014; Huebert et al., 2014). In the kidney, Tanabe et al. suggested that diabetic kidney disease (DKD) progression may involve aberrant capillary formation, and that vascular rarefaction during renal fibrosis could be linked to an imbalance between pro- and anti-angiogenic factors (Tanabe et al., 2020; Tanaka and Nangaku, 2013). Mechanistically, Azad et al. identified FGFR1 as a direct binding partner of YAP1 (Azad et al., 2020), a finding consolidated by Pichol-Thievend et al., who established FGFR1 as an upstream regulator of YAP1 (Pichol-Thievend et al., 2024). From a drug development perspective, FGFR1 represents a well-characterized receptor tyrosine kinase with a defined structure, for which small-molecule inhibitor development is relatively advanced, and several agents have already been approved for clinical use. In contrast, YAP1, as a transcriptional co-activator, lacks a conventional druggable pocket, making the development of specific inhibitors considerably more challenging; most existing candidates remain confined to research tools. Therefore, we propose an alternative “top-down” intervention strategy: by pharmacologically inhibiting FGFR1—an upstream regulator located at the cell membrane and more readily targetable—we can indirectly modulate the activity of its downstream nuclear effector, YAP1. This approach offers a potential novel therapeutic avenue for blocking renal fibrosis.

## 2 Materials and methods

### 2.1 Cell lines and human tissues

Primary human umbilical vein endothelial cells (HUVECs) were obtained from Lonza and authenticated according to a previously described protocol (Chandel et al., 2024). The cells were cultured in complete endothelial cell growth medium (ScienCell; #1001) supplemented with 5% fetal bovine serum (ScienCell; #0025), 1% endothelial cell growth supplement (ScienCell; #1052), and 1% penicillin/streptomycin solution (ScienCell; #0503). For experiments, HUVECs were plated onto six-well plates and maintained at 37 °C under a humidified atmosphere of 5% CO_2_. Human renal tissue samples utilized in this study were collected in accordance with the ethical principles of the Declaration of Helsinki, following approval from the Ethics Committee of Henan Provincial People’s Hospital (Approval No. 2019–06). Written informed consent was obtained from all participants. Specimens designated for the experimental group comprised fibrotic renal parenchyma from patients with clinically and pathologically confirmed chronic kidney disease (CKD) stages 3–5. Control tissues were procured from histologically normal renal parenchyma located more than 5 cm from the tumor margin in surgical resection specimens, all of which were pathologically verified to be free from tumor infiltration and significant pathological alteration.

### 2.2 Cell treatment

HUVECs were cultured with the medium replenished every 48 h to maintain optimal viability. For routine subculture, cells were washed with phosphate-buffered saline (PBS; Solarbio, #P1020), detached with 0.05% trypsin (Solarbio, #T1350; diluted in PBS) for 2 min, and the enzymatic reaction was terminated by adding complete endothelial cell growth medium. HUVECs from passages 4-8 were used for all subsequent experiments.

To establish an *in vitro* fibrosis model, cells were first synchronized by serum-free starvation for 12 h and then stimulated for 24 h with recombinant human TGF-β (Novoprotein Biotech, #CA59; 0, 2.5, 7.5, or 22.5 ng/ml; reconstituted in distilled water). In the subsequent treatment phase, to mimic the pathological microenvironment of persistent TGF-β stimulation as observed *in vivo*, cells were co-treated for 12 h with the FGFR1 inhibitor PD173074 (MedChemExpress, #HY-10321; 0, 10, 30, or 90 nM) in the continued presence of the corresponding TGF-β concentrations. PD173074 was dissolved in dimethyl sulfoxide (DMSO) as a stock solution, with the final DMSO concentration maintained below 0.1% in all cultures to preclude nonspecific cytotoxic effects.

### 2.3 Flow cytometry

For immunophenotypic analysis, cells were harvested and stained for 30 min on ice in the dark using a fluorescein-conjugated mouse anti-human CD31 monoclonal antibody (BD Biosciences, #560984) along with its matched isotype control. After staining, cells were washed twice with PBS to eliminate unbound antibodies, followed by fixation with 4% paraformaldehyde solution. Flow cytometric analysis was performed using a BD FACS AriaIII flow cytometer, with a minimum of 10,000 valid events collected per sample. Data were analyzed via FlowJo V10 software, and HUVECs purity was confirmed when the CD31-positive cell percentage exceeded 99%.

### 2.4 Plasmid construction

To generate shRNA expression constructs directed against FGFR1 and YAP1, the coding sequences (CDS) of human FGFR1 (GenBank: NM_023110.3) and YAP1 (GenBank: NM_001130145.3) were obtained from the NCBI database. Two specific shRNA sequences (GC content 40%–60%) targeting the CDS of each gene were designed with SnapGene 6.0 software. The corresponding oligonucleotides were annealed to generate double-stranded DNA (dsDNA) inserts, which were then ligated into the pLKO.1 vector (Tsingke Biotechnology) using AgeI(New England Biolabs,#R3552L) and EcoRI(New England Biolabs,#R00101V) restriction sites. All plasmid constructs were validated by restriction enzyme digestion and commercial DNA sequencing (Tsingke Biotechnology). The primer sequences for shYAP1 and shFGFR1 are provided in Supplementary Table S1.

### 2.5 Establishment of stable knockdown cell lines

To generate endothelial models with stable knockdown of FGFR1 and YAP1, HUVECs were transduced with lentiviral vectors expressing short hairpin RNAs (shRNAs) targeting the respective human transcripts. A non-targeting scrambled shRNA (shNC) served as the negative control; bioinformatic analysis confirmed its lack of significant homology to the human genome. The shRNA target sequences are listed in Supplementary Table S2. For lentivirus production, HEK293T cells were co-transfected with the shRNA transfer plasmids and the packaging plasmids psPAX2 and pMD2. G (Sangon Biotech,#A338972,#A338961) using Lipo6000 transfection reagent (Beyotime Biotechnology, #C0526). Viral supernatants were harvested at 48 and 72 h post-transfection, concentrated by ultracentrifugation, and stored at −80 °C. Subconfluent HUVECs (∼50–60% confluence) were then transduced with the viral particles. Stable polyclonal populations were selected by treatment with 3 μg/mL puromycin (MedChemExpress, #HY-K1057) for 48 h. Knockdown efficiency was confirmed at the mRNA level by quantitative PCR and at the protein level by Western blot analysis.

### 2.6 Animal

All mouse studies were conducted according to applicable ethical standards and received approval from the Institutional Animal Care and Use Committee at Zhengzhou University. Eight-week-old male wild-type C57BL/6J mice were supplied by GemPharmatech Co., Ltd. (Jiangsu, China). The VECad-Cre (+/+) and YAP1-floxed mouse lines were obtained from Cyagen Biosciences. Additionally, 12 week-old male db/db mice, along with their control db/m mice, were obtained from GemPharmatech Co., Ltd. to establish a type 2 diabetes model. All mice were housed in a specific pathogen-free (SPF) facility under controlled conditions: a 12 h light/dark cycle, ambient temperature of 22 °C–25 °C, and relative humidity maintained at 45%–55%.

### 2.7 Establishment of unilateral ureteral obstruction (UUO) model

Male C57BL/6J mice (8 weeks old) were randomly divided into three groups (n = 6 per group): sham-operated group, UUO group, and UUO + PD173074 treatment group. Mice were anesthetized via intraperitoneal injection of tribromoethanol/tert-amyl alcohol (Macklin, #T903147,#A800283), and the surgical area was shaved and disinfected. Animals were placed in right lateral recumbency to expose the left surgical field. A 1 cm longitudinal incision was made approximately 1 cm below the left costal margin and 1 cm lateral to the spine. After dissecting through the muscle layers, the abdominal cavity was entered, and the intestines were gently retracted to expose the left ureter. The ureter was doubly ligated at two points (proximal and distal segments) using 5–0 absorbable sutures and transected between the ligatures to prevent recanalization. The muscle and skin incisions were sutured layer by layer. Sham-operated mice underwent the same procedure without ureteral ligation or transection. Postoperative health status and wound healing were closely monitored. On day 10 after surgery, mice were euthanized, and kidney tissues were collected. Successful induction of renal fibrosis was confirmed by gross observation of hydronephrosis and histological evaluation of collagen deposition using Masson’s trichrome and Sirius Red staining. Genetically modified mice were subjected to the same surgical protocol (n = 6 per group). All animal experiments were approved by the Institutional Animal Care and Use Committee and conducted in accordance with relevant ethical guidelines.

### 2.8 Histological staining of renal tissues

Renal tissues were immediately fixed in 4% paraformaldehyde for 48 h after collection, followed by paraffin embedding. Serial sections were cut at 3 μm thickness and subjected to hematoxylin and eosin (H&E), Masson’s trichrome, and Sirius Red staining. All staining procedures were performed by Wuhan Servicebio Technology Co., Ltd. following standardized protocols.

### 2.9 Serum creatinine measurement

Prior to the assay, mice were anesthetized, and all facial vibrissae were carefully trimmed to prevent subsequent hemolysis of blood samples through contact with the whiskers. Blood was collected using the retro-orbital venous plexus method. The specific procedure was as follows: the head and dorsal neck skin of the mouse were gently secured with the thumb and index finger of the right hand, while mild pressure was applied to both sides of the neck to temporarily impede venous return, causing sufficient protrusion of the eyeball. The eyeball was then rapidly enucleated using curved forceps, and the vertically flowing blood was collected into a prepared centrifuge tube. To obtain an adequate blood sample, gentle pressure could be applied to the precordial region of the mouse with the left hand during collection to promote cardiac contraction and increase blood flow. The collected whole blood was allowed to clot completely at room temperature for 2–3 h, followed by centrifugation at 4 °C and 3,000 rpm for 15 min. The upper light-yellow serum layer was carefully aspirated for subsequent use. Serum creatinine levels were measured using a commercial creatinine assay kit (Creatinine Assay Kit, sarcosine oxidase method; #C011-2–1; Nanjing Jiancheng Bioengineering Institute) in strict accordance with the manufacturer’s instructions. All animal experimental protocols were reviewed and approved by the Ethics Committee and complied with relevant animal welfare guidelines.

### 2.10 Immunofluorescence staining protocol

To compensate for the challenging staining of certain sensitive epitopes and to precisely observe signal localization, renal tissues were divided and processed in parallel. One portion was fixed in 4% paraformaldehyde for 48 h and subsequently embedded in paraffin, while the other portion was directly embedded in OCT (SAKURA,#4583) compound, rapidly frozen at −80 °C, and used for cryosectioning. Paraffin sections (3 µm thick) were deparaffinized in xylene, rehydrated through a graded ethanol series, and subjected to microwave-assisted antigen retrieval in citrate buffer (10 mM, pH 6.0). All sections were blocked with 5% BSA in PBS for 1 h at room temperature. Cryosections (3 µm thick) were rehydrated at room temperature and blocked under identical conditions. Both types of sections were incubated overnight at 4 °C with the following primary antibodies: anti-YAP1 (1:100; Abcam, #AB205270), anti-VE-cadherin (1:100; R&D Systems, #AF1002), anti-FGFR1 (1:100; Cell Signaling Technology, #9740S), anti-Fibronectin (1:200; Proteintech, #15613-1-AP), anti-Desmin (1:200; Abcam, #AB15200), anti-α-SMA (1:200; Proteintech, #14395-1-AP), and anti-Collagen I (1:200; Proteintech, #14695-1-AP). After washing with PBS, the sections were incubated with species-matched Alexa Fluor-conjugated secondary antibodies (1:500; Abcam, #AB150157, #AB150074) for 1 h at room temperature in the dark. Nuclei were counterstained with DAPI (1ug/ml; Leagene, #DA004), and the slides were mounted with an anti-fade medium (Solarbio,#S2100). Negative controls, processed without primary antibodies, were included to confirm staining specificity. All images were acquired using an Olympus fv3000 laser scanning confocal microscope under identical imaging parameters. In human renal tissues, the positive co-localization staining areas of VE-Cadherin with either FGFR1 or YAP1 were all analyzed using ImageJ software. Cryosections demonstrated superior antigen preservation for sensitive epitopes and served as a reliable alternative when paraffin sections yielded suboptimal staining results.

### 2.11 Western blot analysis

For kidney tissues, samples were placed in a centrifuge tube with magnetic beads and homogenized using high-efficiency RIPA lysis buffer (Solarbio, #R0010) containing 1 mM PMSF. Cultured cells were directly lysed with an equal volume of the same buffer. After incubation on ice for 20 min, the lysates were centrifuged at 13,000 × g for 5 min at 4 °C. The supernatant was collected by removing the precipitate. Protein concentration was determined using a BCA Protein Assay Kit (Beyotime, #P0010S) according to the manufacturer’s instructions, and equal amounts of protein were loaded for subsequent analysis. Proteins were separated by SDS-PAGE (NewCell&Molecular Biotech Co., Ltd.,#PN1012,#PN1013) and transferred onto PVDF membranes. The membranes were then blocked at room temperature for 1 h with 5% skim milk in TBST buffer (Servicebio, #G0004). Subsequently, the membranes were incubated overnight at 4 °C on a shaker with the following specific primary antibodies: FGFR1 (1:1000; Cell Signaling Technology, #9740S), YAP1 (1:2000; Proteintech, #13584-1-AP), α-SMA (1:8000; Proteintech, #14395-1-AP), Fibronectin (1:1000; ZENBIO, #250073), Vimentin (1:1000; Abcam, #AB92547),p-VE-cadherin (1:500; Affinity, #AF3265),p-ERK (1:500; Cell Signaling Technology, #9107T),VCAM1(1:1000; Starter, #S0B0407), IL-6 (1:1000; Abcam, #AB259341), GAPDH (1:1000; Servicebio,#GB11002-100). This was followed by incubation with HRP-conjugated goat anti-mouse IgG (1:10000; Servicebio, #GB23301-100) or HRP-conjugated goat anti-rabbit IgG (1:10000; Servicebio, #GB23303-100) on a shaker at room temperature for 1 h. Protein signals were detected using the Omni-ECL Femto Light Chemiluminescence Kit (EpiZyme, #SQ201), and band intensities were quantified by densitometry analysis with ImageJ software. The data were normalized to GAPDH as the loading control. For target proteins with similar molecular weights, the membranes were stripped using a rapid stripping buffer for Western blot antibodies (EpiZyme,#PS107S) and then re-probed with the corresponding primary and secondary antibodies following the same procedure described above.

### 2.12 Quantitative real-time PCR analysis

Total RNA was isolated from mouse renal tissues and human umbilical vein endothelial cells (HUVECs) using the FastPure Cell/Tissue Total RNA Isolation Kit V2 (Vazyme, #RC112-01) according to the manufacturer’s instructions. The extracted RNA was resuspended in DEPC-treated water (Thermo Fisher Scientific, #750023). RNA concentration and purity were determined using a NanoDrop™ OneC spectrophotometer (Thermo Fisher Scientific), with all samples yielding A260/A280 ratios between 1.8 and 2.1. For cDNA synthesis, 1 µg of total RNA was reverse-transcribed using the PrimeScript RT reagent kit (Takara, #RR047A) for qPCR, following the manufacturer’s protocol. Quantitative PCR (qPCR) was performed using SYBR Green Master Mix (Vazyme, #Q511-02) on an Applied Biosystems 7,500 Real-Time PCR System. The thermal cycling protocol consisted of an initial denaturation at 95 °C for 30 s, followed by 40 cycles of 95 °C for 5 s and 60 °C for 30 s. All primer sequences are listed in Supplementary Table S3. GAPDH was used as the endogenous reference gene, and relative mRNA expression levels were calculated using the 2^(–ΔΔCt) method.

### 2.13 Statistical analysis

All data are presented as mean ± SD. Comparisons between two groups were performed using either unpaired or paired two-tailed t-tests based on the experimental design. For comparisons among three or more groups, one-way analysis of variance (ANOVA) was applied. If the overall comparison showed statistical significance (F-test, P < 0.05) and the assumption of homogeneity of variance was met, Tukey’s test was further used for post-hoc pairwise comparisons. All statistical analyses were conducted using GraphPad Prism software (version 8.0), and a P-value <0.05 was considered statistically significant.

## 3 Result

### 3.1 Coordinated upregulation of FGFR1 and YAP1 in endothelial cells of human fibrotic kidneys

**FIGURE 1:**
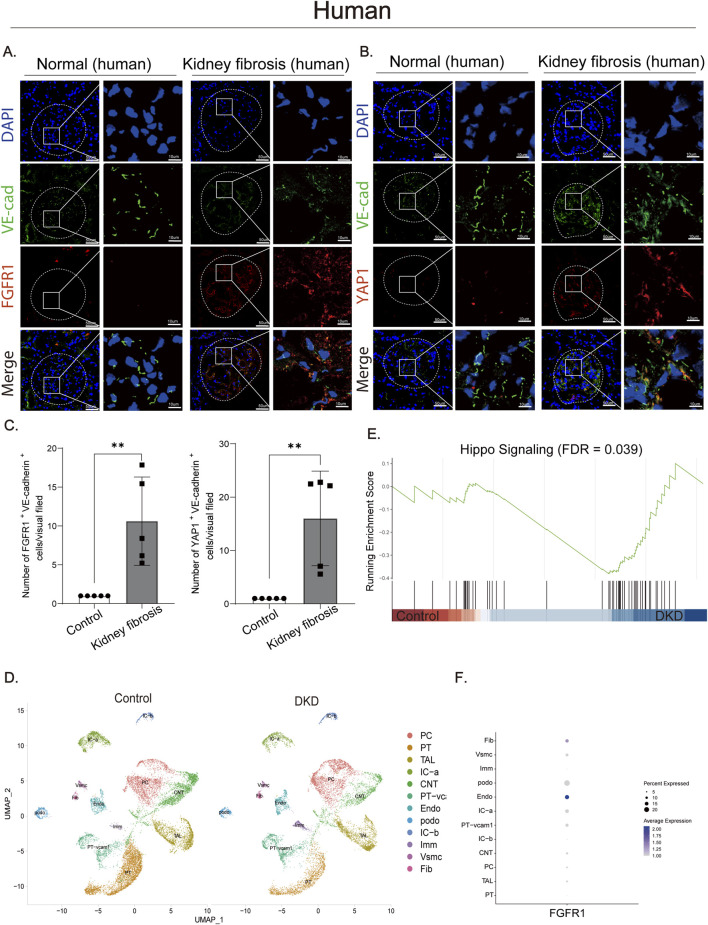
The FGFR1/YAP1 axis is upregulated in human renal fibrosis, with single-cell transcriptomic validation and enrichment of Hippo pathway signaling in DKD. **(A)** Immunofluorescence micrographs showing co-localization of FGFR1 (red) and VE-cadherin (green) in adjacent non-cancerous (Normal) and renal fibrosis tissues. Bar = 50 μm and 10 μm. Dashed circles outline the glomeruli. **(B)** Immunofluorescence staining comparing co-localization of YAP1 (red) and VE-cadherin (green) between Normal and renal fibrosis tissues. Bar = 50 μm and 10 μm. Dashed circles outline the glomeruli. **(C)** Semi-quantitative immunofluorescence analysis of FGFR1 (left) and YAP1 (right) expression in renal endothelial cells. Both FGFR1 and YAP1 are significantly upregulated in renal fibrosis compared to Normal tissues (normalized to 1 arbitrary unit). n = 5. **(D)** UMAP shows cell population in kidneys from healthy controls and patients with DKD. PC principal cell of collecting duct, PT proximal tubule,TAL thick ascending limb of LoH, IC-a type A intercalated cell of collecting duct, CNT connecting tubule, PT-vc proximal tubule vascular loop cell, Endo endothelial cell, podo podocyte, IC-b type B intercalated cell of collecting duct, Vsmc vascular smooth muscle cell, Fib fibroblast. Data from: GSE131882. **(E)** GSEA shows that the Hippo signaling pathway was enriched in the DKD group *versus* the control group (FDR = 0.039). **(F)** Graphic presentation of single-cell sequencing analysis shows the expression of FGFR1 in different cell populations. Data are presented as mean ± SD. **p < 0.01.

To examine YAP1 and FGFR1 expression in human renal fibrosis, we obtained fibrotic renal tissues from patients with advanced CKD (stages 3–5). Non-fibrotic control tissues were obtained from the tumor-normal junction of nephrectomy specimens from patients with renal cell carcinoma. Histological analysis using H&E, Masson’s trichrome, and PAS staining confirmed significant interstitial fibrosis and inflammatory cell infiltration in fibrotic tissues compared to controls (Supplementary Figure S1A). Immunofluorescence staining revealed a marked intensification of both FGFR1 (Figure 1A) and YAP1 (Figure 1B) signals. (red) within the endothelium of fibrotic tissues compared to controls. Critically, both proteins demonstrated clear co-localization with the endothelial marker VE-cadherin (green), confirming their upregulation in endothelial cells (Figures 1A,B). While FGFR1 expression is not restricted to endothelial cells, its marked upregulation within the VE-cadherin-positive endothelial compartment—quantified by co-localization analysis—indicates a specific role for endothelial FGFR1 during fibrosis. Semi-quantitative analysis of fluorescence intensity demonstrated that the expression levels of YAP1 and FGFR1 in endothelial cells of fibrotic tissues were increased by approximately 15-fold and 10-fold, respectively, compared to the control group (Figure 1C). To address potential bias arising from limited sample size and to further validate the cellular localization of FGFR1, we examined a public single-cell RNA-seq dataset (GSE131882) comparing kidneys from healthy controls and patients with DKD (Figure 1D), a major etiology of CKD. Single-cell transcriptomic profiling revealed that FGFR1 was predominantly enriched in endothelial cell populations (Figure 1F). Consistent with the upregulation of YAP1 observed by immunofluorescence, Gene Set Enrichment Analysis (GSEA) showed significant enrichment of the Hippo signaling pathway in the DKD group compared to controls (Figure 1E). These findings align closely with our immunofluorescence observations and suggest that coordinated activation of the endothelial FGFR1/YAP1 signaling axis represents a critical molecular event in human renal fibrosis, offering a clinical rationale for further investigation into their functional interplay.

### 3.2 Activation of the FGFR1/YAP1 axis mediates TGF-β-induced endothelial fibrosis

**FIGURE 2:**
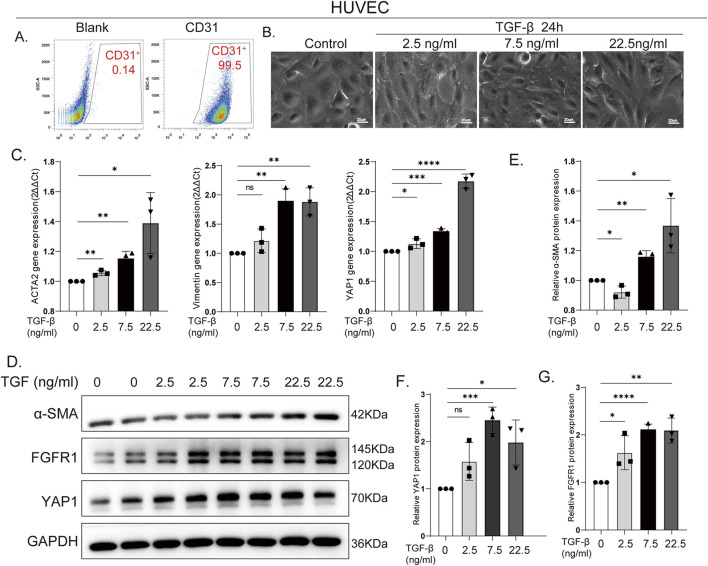
TGF-β induces endothelial cell profibrotic activation via the FGFR1/YAP1 axis. **(A)** Flow cytometry verifying >99% purity of isolated human umbilical vein endothelial cells (HUVECs) by CD31 expression. **(B)** Morphological changes of HUVECs treated with TGF-β (2.5, 7.5, 22.5 ng/ml): concentration-dependent transition from cobblestone to spindle-shaped, smooth muscle cell-like phenotype. Bar = 20 μm. **(C)** qPCR analysis of fibrotic markers (ACTA2, Vimentin) and YAP1 mRNA in TGF-β-stimulated HUVECs. Data are fold change vs. unstimulated control (set as 1). **(D)** Western blot showing TGF-β-induced upregulation of α-SMA, FGFR1, and YAP1 in HUVECs after 24-h treatment (0, 2.5, 7.5, 22.5 ng/ml). Data are fold change vs. unstimulated control (set as 1). **(E–G)** Semi-quantitative Western blot confirming TGF-β-induced upregulation of α-SMA (dose-dependent; **(E)**, FGFR1 **(F)**, and YAP1 **(G)** in HUVECs. n = 3. Data are presented as mean ± SD. *p < 0.05, **p < 0.01, ***p < 0.001, ****p < 0.0001; ns, no significance.

To establish an *in vitro* model of endothelial fibrosis, primary human umbilical vein endothelial cells (HUVECs) were treated with TGF-β. High-purity HUVECs (>95% CD31^+^) (Figure 2A) were stimulated with TGF-β at concentrations of 2.5, 7.5, and 22.5 ng/ml, selected based on previous literature. Morphological analysis revealed that TGF-β induced a concentration-dependent transition of HUVECs from their typical cobblestone morphology into a spindle-shaped, smooth muscle-like phenotype (Figure 2B). At the molecular level, qPCR assays showed that TGF-β stimulation significantly upregulated the mRNA expression of the fibrosis markers ACTA2 and Vimentin, concomitant with an increase in YAP1 mRNA (Figure 2C). This finding was further corroborated by Western blot analysis, which confirmed that treatment with 22.5 ng/ml TGF-β for 24 h markedly enhanced the protein expression of α-SMA, FGFR1, and YAP1 (Figure 2D). Semi-quantitative Western blot analysis showed that the protein expression of α-SMA (Figure 2E), YAP1 (Figure 2F), and FGFR1 (Figure 2G) was significantly upregulated after TGF-β stimulation. Notably, treatment with 22.5 ng/mL TGF-β yielded stable pro-fibrotic model induction, and the upregulation of all target proteins was statistically significant compared with the control group. Consequently, a concentration of 22.5 ng/ml TGF-β was utilized for all subsequent experiments. Collectively, these results demonstrate that TGF-β activates the FGFR1/YAP1 signaling axis in endothelial cells, driving their transition toward a fibrotic phenotype and highlighting a pivotal role for this axis in fibrosis.

### 3.3 The FGFR1 inhibitor PD173074 attenuates TGF-β-induced endothelial-to-mesenchymal transition

**FIGURE 3:**
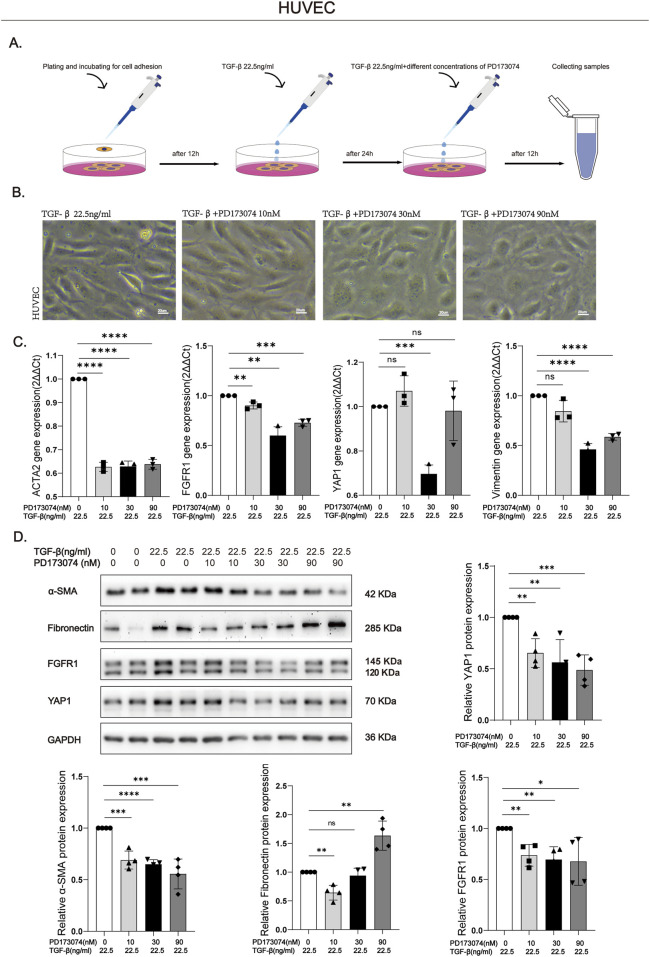
PD173074, an FGFR1 inhibitor, mitigates TGF-β-induced EndMT via targeting the FGFR1/YAP1 axis **(A)** Experimental protocol: HUVECs were seeded in 35 mm dishes, adhered for 12 h, then treated with 22.5 ng/ml TGF-β for 24 h to induce fibrosis, followed by intervention with 22.5 ng/ml TGF-β plus PD173074 (0, 10, 30, 90 nM). **(B)** Phase-contrast images showing PD173074 (30, 90 nM) reversed TGF-β-induced morphological transition from spindle-shaped to cobblestone-like phenotype. Bar = 20 μm **(C)** qPCR analysis of FGFR1, YAP1, and EndMT markers (ACTA2, Vimentin) mRNA in HUVECs under indicated conditions. Data normalized to GAPDH, presented as fold change vs. untreated control (set to 1). n = 3. **(D)** Western blot confirming PD173074-induced downregulation of FGFR1, YAP1, and α-SMA, consistent with GAPDH-normalized semi-quantitative data (fold change vs. untreated control, set to 1). n = 4. Data are presented as mean +SD. *p < 0.05, **p < 0.01, ***p < 0.001, ****p < 0.0001; ns, no significance.

To investigate the functional role of FGFR1, HUVECs undergoing TGF-β stimulation were treated with the specific FGFR1 inhibitor PD173074 (experimental scheme outlined in Figure 3A). To maintain consistent drug activity, the culture medium was replenished with fresh TGF-β and PD173074 after 24 h of treatment. Morphological assessment demonstrated that co-treatment with PD173074 (at 30 and 90 nM) effectively suppressed the TGF-β-induced morphological shift, restoring the characteristic polygonal, cobblestone-like endothelial architecture (Figure 3B). qPCR analysis indicated that PD173074 administration downregulated the mRNA expression of both FGFR1 and its downstream effector YAP1. Concurrently, the transcript levels of EndMT-associated markers, ACTA2 and Vimentin, were significantly reduced (Figure 3C). This attenuation was further confirmed at the protein level, where PD173074 treatment decreased the expression of FGFR1, YAP1, and α-SMA (Figure 3D). Collectively, these findings demonstrate that pharmacological inhibition of FGFR1 attenuates TGF-β-driven EndMT at least in part by suppressing YAP1 signaling, supporting FGFR1 as a key upstream regulator of YAP1 and highlighting its potential for future therapeutic development.

### 3.4 Genetic silencing of FGFR1 or YAP1 attenuates EndMT and reveals downstream mechanistic insights

**FIGURE 4:**
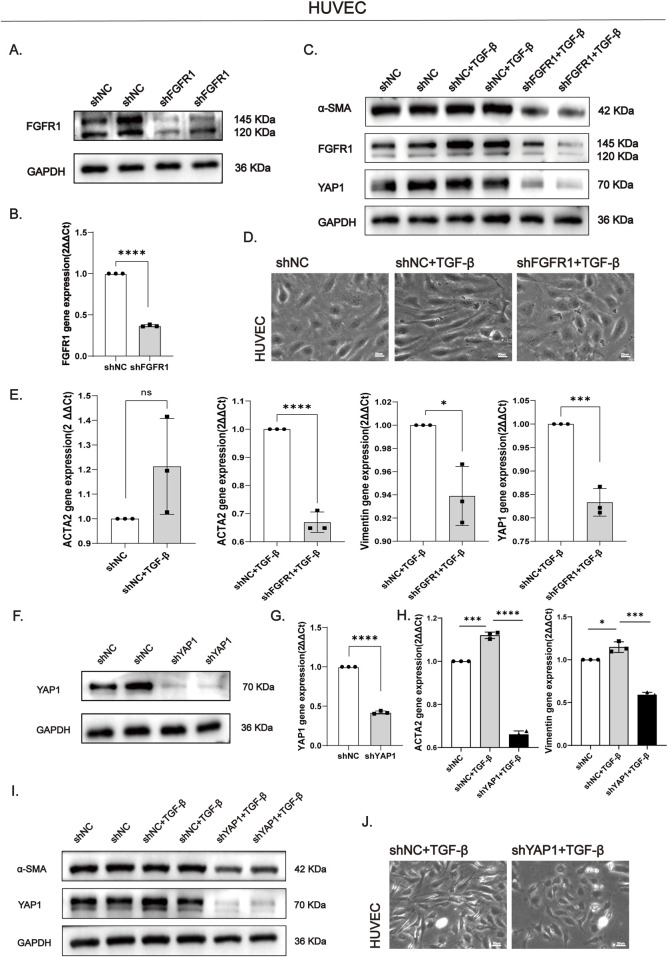
FGFR1 or YAP1 knockdown attenuates EndMT **(A,B)** Validation of FGFR1 knockdown efficiency in HUVECs at the protein **(A)** and mRNA **(B)** levels. **(C)** Western blot analysis demonstrated that TGF-β induces the upregulation of α-SMA, YAP1, and FGFR1 expression in shNC cells, while knockdown of FGFR1 inhibits this effect. **(D)** Morphological reversion of HUVECs from spindle-shaped (TGF-β-induced) to cobblestone-like after FGFR1 knockdown. Bar = 20 μm. **(E)** qPCR analysis showing TGF-β-induced upregulation of ACTA2, YAP1, ACTA2 and Vimentin in control cells, which was suppressed by FGFR1 knockdown. **(F,G)** Validation of YAP1 knockdown efficiency at the protein **(F)** and mRNA **(G)** levels. **(H)** qPCR analysis of ACTA2 and Vimentin mRNA confirming TGF-β-induced fibrosis and suppression by YAP1 knockdown. **(I)** Western blot analysis demonstrated that YAP1 knockdown significantly suppressed the TGF-β-induced upregulation of α-SMA and YAP1 expression. **(J)** Morphological reversion of HUVECs to cobblestone-like after YAP1 knockdown.​ Bar = 20 μm. All data are presented as mean +SD (n = 3). *p < 0.05, **p < 0.01, ***p < 0.001, ****p < 0.0001; ns, no significance. mRNA data were normalized to GAPDH or β-actin and expressed as fold change vs. scrambled control (set to 1).

To exclude potential off-target effects of the pharmacological inhibitor and definitively establish the core function of FGFR1, we performed specific knockdown of the FGFR1 gene in HUVECs using shRNA. The knockdown efficiency was verified by Western blot (Figure 4A) and qPCR (Figure 4B). Under TGF-β stimulation, FGFR1 knockdown not only markedly suppressed the mRNA and protein expression of EndMT markers (ACTA2/α-SMA, Vimentin) but also led to a concomitant reduction in its downstream effector, YAP1, at both the translational (Figure 4C) and transcriptional (Figure 4E) levels. This genetic intervention also reversed the TGF-β-induced spindle-like morphology, restoring the endothelial cobblestone appearance (Figure 4D). These results directly identify YAP1 as a critical downstream effector of FGFR1 signaling in EndMT, forming a coherent functional axis. To further validate the necessity of YAP1, we knocked down its expression in HUVECs. The knockdown efficiency was verified by Western blot (Figure 4F) and qPCR (Figure 4G). As anticipated, YAP1 deficiency effectively counteracted the TGF-β-induced EndMT phenotype, reverting the cells to a cobblestone-like morphology (Figure 4J) and significantly reducing the expression of α-SMA protein (Figure 4I) as well as ACTA2 and Vimentin mRNA (Figure 4H). This confirms the pivotal role of YAP1 in driving EndMT and solidifies the central role of the FGFR1/YAP1 signaling axis in renal fibrosis, thereby providing a molecular foundation for future *in vivo* intervention strategies.

**FIGURE 5:**
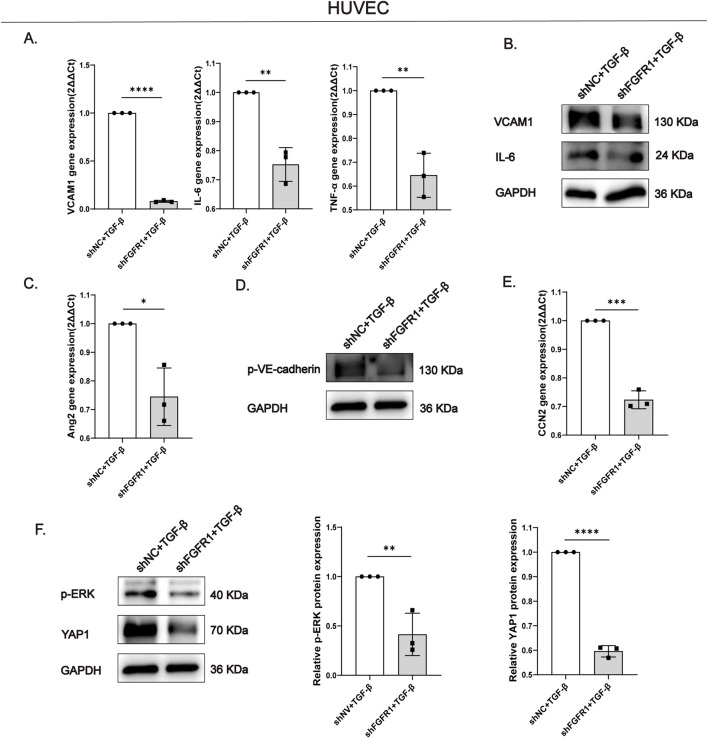
FGFR1 knockdown attenuates inflammation and endothelial dysfunction and reveals underlying mechanisms **(A)** qPCR analysis showed that FGFR1 knockdown reduced the mRNA levels of VCAM1, IL-6, and TNF-α under TGF-β stimulation. **(B)** Western blot analysis confirmed that FGFR1 knockdown decreased the protein expression of VCAM1 and IL-6 under TGF-β stimulation. **(C)** Under TGF-β stimulation, FGFR1 knockdown downregulated Ang2 mRNA expression compared with the shNC group. **(D)** FGFR1 knockdown decreased p-VE-cadherin protein expression under TGF-β stimulation relative to the shNC group. **(E)** Under TGF-β stimulation, FGFR1 knockdown reduced CCN2 mRNA expression compared with the shNC group. **(F)** Western blot analysis revealed that FGFR1 knockdown downregulated p-ERK and YAP1 protein levels under TGF-β stimulation relative to the shNC group. All data are presented as mean +SD (n = 3). *p < 0.05, **p < 0.01, ***p < 0.001, ****p < 0.0001; ns, no significance. mRNA data were normalized to GAPDH and expressed as fold change vs. scrambled control (set to 1).

We next investigated the broader impact of FGFR1 knockdown beyond EndMT. Quantitative real-time PCR analysis revealed that FGFR1 silencing significantly reduced the mRNA expression levels of vascular cell adhesion molecule 1 (VCAM-1), a key mediator of immune cell adhesion, as well as the pro-inflammatory cytokines interleukin-6 (IL-6) and tumor necrosis factor-α (TNF-α) in HUVECs under TGF-β stimulation (Figure 5A). Consistently, Western blot analysis confirmed that VCAM-1 and IL-6 protein levels were also markedly downregulated following FGFR1 knockdown (Figure 5B). Furthermore, FGFR1 deficiency attenuated endothelial injury, as evidenced by the transcriptional downregulation of angiopoietin-2 (Ang2), a molecule associated with endothelial dysfunction (Figure 5C). Notably, the levels of tyrosine-phosphorylated VE-cadherin (p-VE-cadherin), a marker of disrupted endothelial tight junctions, were also significantly reduced upon FGFR1 knockdown (Figure 5D). Furthermore, FGFR1 knockdown resulted in a significant reduction in the mRNA levels of connective tissue growth factor (CCN2) (Figure 5E). As a pro-fibrotic paracrine mediator predominantly derived from endothelial cells, CCN2 is known to stimulate fibroblast activation and facilitate extracellular matrix accumulation. The observed decrease in CCN2 expression implies that loss of FGFR1 may alleviate the pro-fibrotic milieu by interfering with endothelial-to-fibroblast paracrine communication.

To delineate the molecular mechanism by which FGFR1 regulates YAP1 expression, we examined the phosphorylation status of extracellular signal-regulated kinase (ERK), a canonical downstream signaling node of FGFR1. Western blot analysis demonstrated that FGFR1 knockdown substantially inhibited TGF-β-induced ERK phosphorylation (p-ERK) (Figure 5F). This result suggests that FGFR1 mediates YAP1 upregulation, at least in part, through activation of the ERK signaling pathway.

Collectively, these findings demonstrate that FGFR1 knockdown not only suppresses EndMT but also ameliorates endothelial dysfunction, inflammatory activation, and potential paracrine signaling between endothelial cells and fibroblasts. The identification of the FGFR1-ERK-YAP1 signaling axis provides mechanistic insight into the link between FGFR1 activation and the downstream transcriptional programs driving renal fibrosis.

### 3.5 Endothelial-specific deletion of YAP1 attenuates renal fibrosis in vivo

**FIGURE 6:**
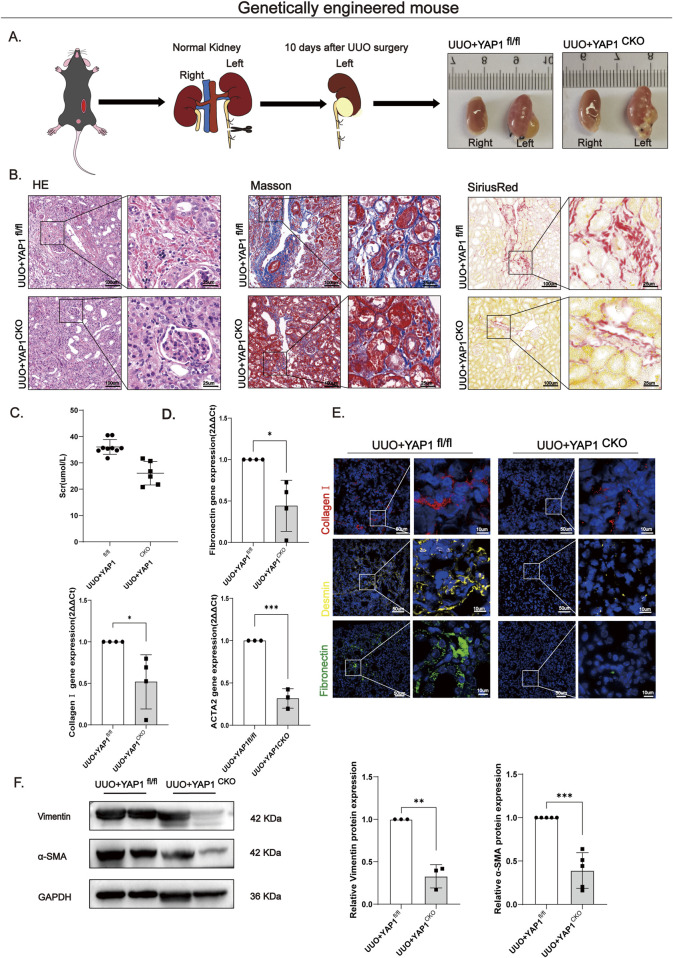
Endothelial YAP1 deletion mitigates renal fibrosis *in vivo*. **(A)** Gross morphology of kidneys from YAP1fl/fl and YAP1 conditional knockout (CKO) mice after unilateral ureteral obstruction (UUO). Both genotypes showed severe hydronephrosis and parenchymal atrophy in the obstructed left kidneys. **(B)** H&E, Masson’s trichrome, and Sirius Red staining revealed that YAP1 CKO mice had reduced eosinophilic deposits **(H&E)**, decreased collagen fiber accumulation (Masson’s trichrome), and preserved renal architecture with diminished fibrosis (Sirius Red) compared to YAP1fl/fl controls. Bar = 100 μm and 25 μm. **(C)** Serum creatinine levels post-UUO indicated improved renal function in YAP1 CKO mice (n ≥ 5). **(D)** qPCR analysis of fibrotic genes (ACTA2, Fibronectin, Collagen I in UUO kidneys showed downregulated mRNA levels in YAP1 CKO *versus* YAP1fl/fl mice (GAPDH-normalized; fold change vs. control = 1; n ≥ 3). **(E)** Immunofluorescence staining demonstrated reduced expression of extracellular matrix proteins (Fibronectin, Collagen I and mesenchymal marker Desmin in YAP1 CKO kidneys compared to controls. Bar = 50 μm and 10 μm. **(F)** Western blot and semi-quantification (GAPDH-normalized; n ≥ 3; fold change vs. control = 1) showed reversed UUO-induced upregulation of α-SMA and Vimentin proteins in YAP1 CKO kidneys. Data are presented as mean ± SD. *p < 0.05, **p < 0.01, ***p < 0.001, ****p < 0.0001; ns, no significance.

While our *in vitro* findings identified a critical role for the FGFR1/YAP1 signaling axis in TGF-β-induced EndMT, its causal contribution and therapeutic relevance in renal fibrosis *in vivo* required direct validation. To this end, we generated endothelial-specific YAP1 knockout mice (YAP1 CKO; VECad-Cre+/−, YAP1fl/fl) by crossing VECad-Cre mice with YAP1 floxed mice, using VECad-Cre−/−, YAP1fl/fl littermates as controls (Supplementary Figure S2A,B). Tamoxifen induction (3-day-on/3-day-off for 3 cycles) was followed by unilateral ureteral obstruction (UUO) surgery 7 days post the last injection (Supplementary Figure S2C). Gross morphological examination of UUO kidneys revealed a loss of the normal bean-shaped architecture, resulting in thin-walled, translucent, fluid-filled cystic structures with parenchymal atrophy and hydronephrosis in both genotypes, showing no overt macroscopic differences (Figure 6A). However, detailed histopathological analysis uncovered significant mitigation of fibrosis in YAP1 CKO mice. In YAP1 fl/fl controls, H&E staining displayed substantial renal structural disruption, inflammatory infiltration, and deposition of amorphous pink material. Sirius Red staining demonstrated disorganized renal architecture with enhanced fibrotic deposition, and Masson’s Trichrome staining confirmed extensive blue collagen fiber accumulation in glomeruli and the interstitium. These pathological features were markedly ameliorated in YAP1 CKO mice (Figure 6B). Functionally, YAP1 CKO mice exhibited significantly lower serum creatinine levels compared to controls (Figure 6C), indicating preserved renal function. At the molecular level, qPCR analysis of kidney tissues from YAP1 CKO mice showed significant downregulation of fibrosis-related genes, including ACTA2, Fibronectin, and Collagen I (Figure 6D). Immunofluorescence staining further demonstrated markedly reduced deposition of Fibronectin, Collagen I, and Desmin (Figure 6E). Correspondingly, Western blot and semi-quantitative analysis confirmed the significant downregulation of the myofibroblast activation markers Vimentin and α-SMA in the YAP1 CKO group (Figure 6F). Collectively, these *in vivo* results establish endothelial YAP1 as a pivotal driver of renal fibrosis. Its specific ablation attenuates UUO-induced renal fibrosis and preserves renal function by suppressing myofibroblast activation and ECM deposition, thereby providing pivotal genetic evidence for targeting the FGFR1/YAP1 signaling axis.

### 3.6 Pharmacological targeting of the FGFR1/YAP1 axis ameliorates renal fibrosis *in vivo*

**FIGURE 7:**
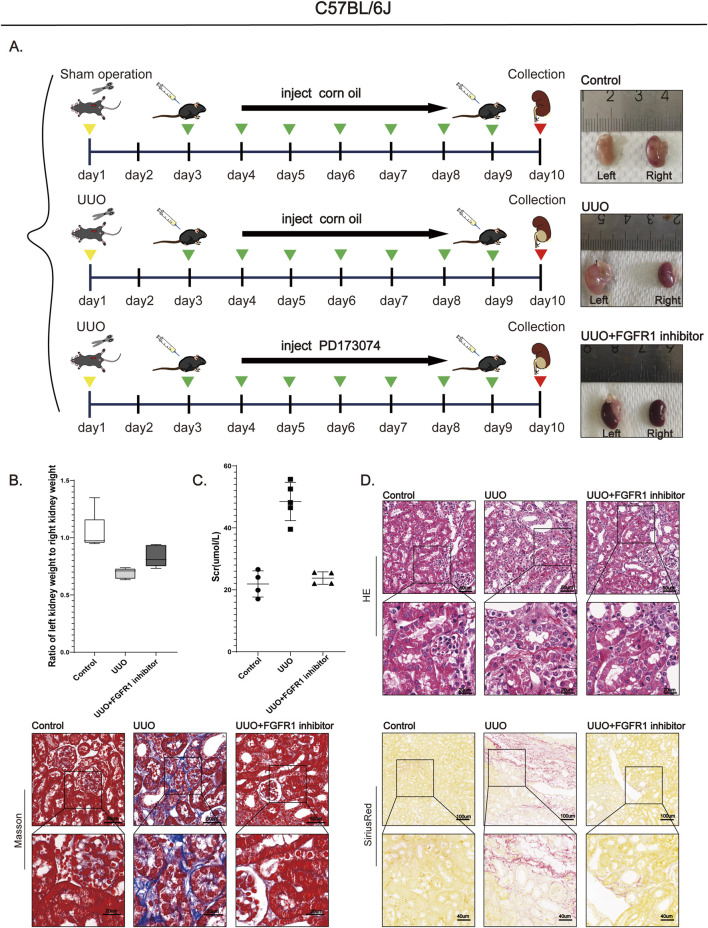
FGFR1 inhibitor PD173074 ameliorates UUO-induced renal atrophy, dysfunction, and tissue fibrosis. **(A)** Experimental timeline and gross renal morphology of sham-operated (control), UUO + vehicle (UUO), and UUO + PD173074 groups. **(B)** Left/right dry kidney weight ratio across experimental groups. **(C)** Serum creatinine levels in sham, UUO, and UUO + PD173074 mice. **(D)** Histological staining (H&E, Masson’s trichrome, Sirius Red): The UUO group showed typical fibrotic features (amorphous eosinophilic deposits, increased collagen, disrupted renal architecture) vs. controls (confirming successful modeling); PD173074 alleviated these changes. Bar = 50 μm and 20 μm, except Sirius Red staining (100 μm and 40 μm).

**FIGURE 8:**
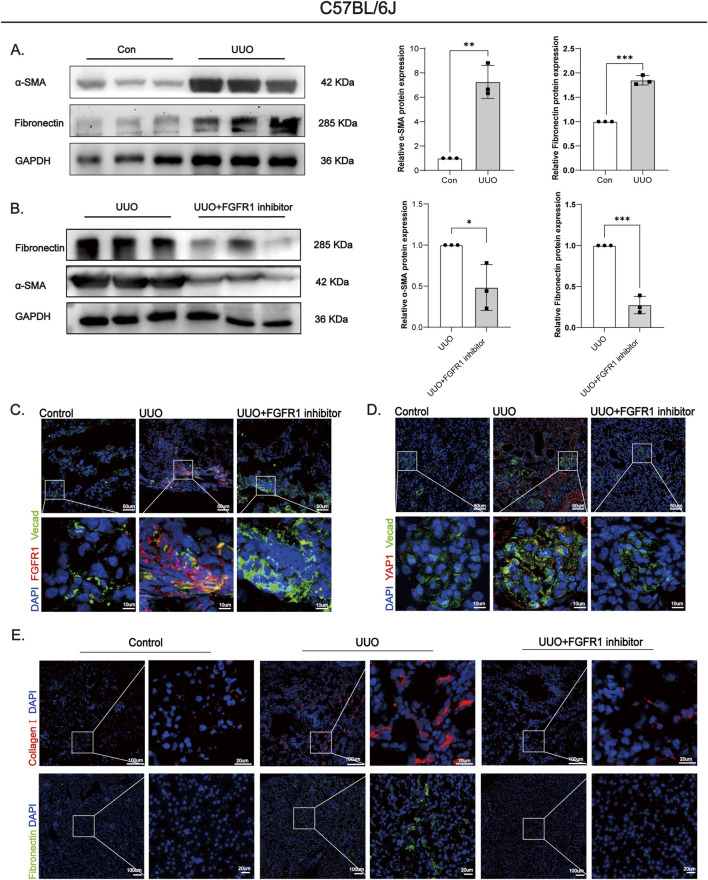
FGFR1 inhibitor PD173074 attenuates molecular markers of fibrosis induced by UUO. **(A)** Western blot and semi-quantification (GAPDH-normalized; fold change vs. control = 1): UUO upregulated α-smooth muscle actin (α-SMA) and Fibronectin in mouse renal tissues vs. sham. **(B)** Western blot and semi-quantification (GAPDH-normalized; fold change vs. UUO = 1): PD173074 reversed UUO-induced α-SMA and Fibronectin upregulation. **(C,D)** Immunofluorescence (FGFR1/vascular endothelial cadherin (VE-cadherin), YAP1/VE-cadherin; red/green): UUO-induced FGFR1 **(C)** and Yes-associated protein 1 (YAP1) **(D)** upregulation in renal endothelial cells was inhibited by PD173074. Bar = 50 μm and 10 μm **(E)** Immunofluorescence: UUO-induced upregulation of extracellular matrix components (Fibronectin, Collagen I in renal tissues was downregulated by PD173074. Bar = 100 μm and 20 μm. All data are presented as mean +SD (n ≥ 3). *p < 0.05, **p < 0.01, ***p < 0.001, ****p < 0.0001; ns, no significance.

Building upon the genetic evidence establishing endothelial YAP1 as a key driver of renal fibrosis (Section 3.5), and recognizing the challenges of targeting transcriptional coactivators therapeutically, we next evaluated the pharmacological potential of its upstream regulator FGFR1. Given that FGFR1, a receptor tyrosine kinase, presents a more tractable drug target, we evaluated the therapeutic potential of its specific inhibitor PD173074 in a murine unilateral ureteral obstruction (UUO) model. Wild-type C57BL/6J mice underwent UUO surgery and received intraperitoneal administration of PD173074, with experimental timeline outlined in Figure 7A. Gross morphological examination revealed typical hydronephrotic changes–including renal enlargement, parenchymal atrophy, and capsular thinning–in both UUO vehicle and PD173074-treated groups. However, quantitative assessment of renal parenchymal preservation demonstrated a significantly higher kidney weight ratio (ipsilateral/contralateral) in PD173074-treated mice compared to vehicle controls (Figure 7B), indicating amelioration of renal atrophy. Functionally, PD173074 treatment significantly suppressed the UUO-induced elevation in serum creatinine, restoring it to levels comparable to sham-operated animals (Figure 7C). Histopathological evaluation further corroborated the therapeutic efficacy of FGFR1 inhibition. While H&E staining of vehicle-treated UUO kidneys showed severe tubular injury–characterized by tubular dilation, epithelial vacuolization, and acellular eosinophilic casts–PD173074 administration markedly improved structural integrity. Consistently, Masson’s trichrome and Sirius Red staining revealed substantial collagen deposition in UUO controls, which was significantly reduced upon PD173074 treatment (Figure 7D). At the molecular level, Western blot analysis revealed significant upregulation of the fibrotic markers α-SMA and fibronectin in renal tissues from UUO model mice relative to sham-operated controls (Figure 8A). Conversely, treatment of UUO mice with the FGFR1-specific inhibitor PD173074 significantly reversed the aberrant overexpression of these fibrotic markers (Figure 8B). Immunofluorescence staining demonstrated co-upregulation of FGFR1 (Figure 8C) and YAP1 (Figure 8D) in endothelial cells of fibrotic kidneys, an effect abolished by PD173074 administration. Concomitantly, PD173074 suppressed the accumulation of extracellular matrix components, including collagen I and fibronectin (Figure 8E).

**FIGURE 9:**
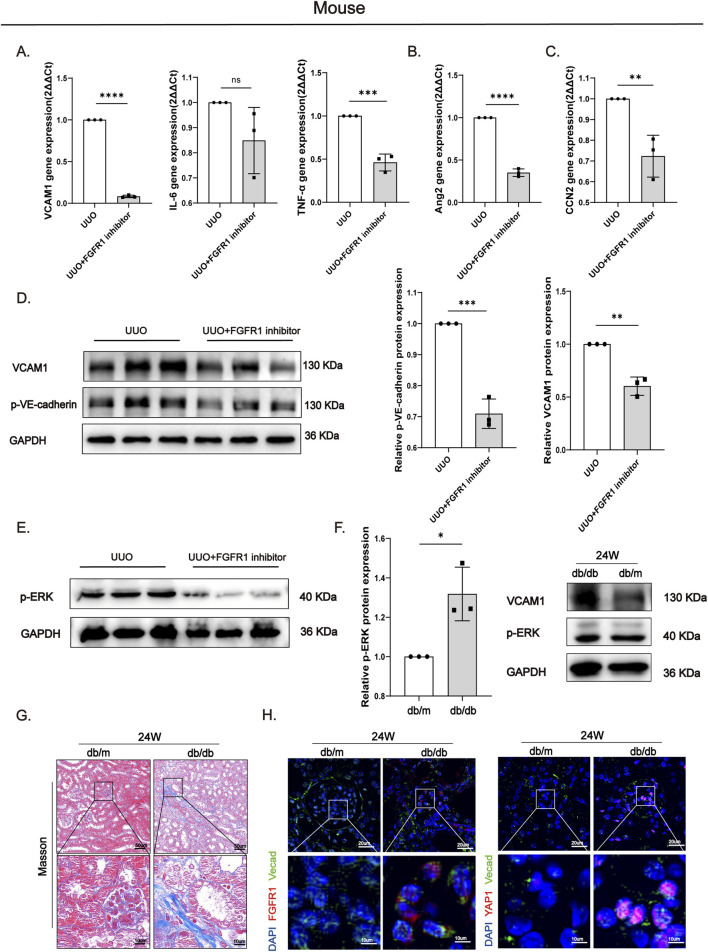
FGFR1 inhibition reduces inflammatory and endothelial permeability markers and suppresses p-ERK/YAP1 signaling in UUO and db/db mice. **(A)** qPCR analysis showed that FGFR1 inhibitor reduced VCAM1, IL-6, and TNF-α mRNA levels in UUO mice. **(B,C)** qPCR results confirmed that Ang2 and CCN2 mRNA levels were decreased in the UUO + FGFR1 inhibitor group. **(D)** Western blot analysis revealed reduced protein levels of VCAM1 and p-VE-cadherin in the UUO + FGFR1 inhibitor group. **(E)** p-ERK protein expression was decreased in the UUO + FGFR1 inhibitor group. **(F)** VCAM1 and p-ERK protein levels were elevated in 24 week db/db diabetic mice compared to db/m controls. **(G)** Masson staining showed marked renal interstitial fibrosis in 24 week db/db mice. **(H)** Immunofluorescence demonstrated increased FGFR1 and YAP1 expression in endothelial cells of 24 week db/db mice. All data are presented as mean +SD (n ≥ 3). *p < 0.05, **p < 0.01, ***p < 0.001, ****p < 0.0001; ns, no significance.

To determine whether the anti-inflammatory and vascular protective effects observed *in vitro* were recapitulated *in vivo*, we evaluated the impact of PD173074 on the renal microenvironment. The results showed that PD173074 administration significantly suppressed both the mRNA and protein levels of the inflammatory factors VCAM1, IL-6, and TNF-α in kidney tissues (Figures 9A,D), indicating that FGFR1 blockade effectively mitigates UUO-induced local inflammation. Concurrently, treatment with PD173074 decreased the expression of Ang2, a marker closely associated with vascular leakage, and significantly reduced the protein level of p-VE-cadherin (Figures 9B,D), suggesting restoration of vascular barrier function. Furthermore, PD173074 treatment led to a significant downregulation of CCN2 mRNA expression (Figure 9C), implying that FGFR1 inhibition may disrupt the paracrine signaling from activated endothelial cells to fibroblasts. At the mechanistic level, PD173074 administration also blocked UUO-induced ERK phosphorylation (Figure 9E), consistent with our *in vitro* observations and further supporting the pivotal role of the FGFR1-ERK-YAP1 signaling axis in the progression of renal fibrosis.

To explore the translational relevance of this signaling axis, we extended our validation to a mouse model of diabetic kidney disease (DKD). Masson’s trichrome staining (Figure 9G) revealed marked renal fibrosis in renal tissues of our established DKD model mice (db/db) relative to healthy control mice(db/m). Compared to healthy controls, DKD mice with established renal fibrosis exhibited significantly elevated expression of the immune cell adhesion molecule VCAM1 and increased levels of phosphorylated ERK in the kidney (Figure 9F). Immunofluorescence staining confirmed the co-upregulation of FGFR1 and YAP1 specifically in endothelial cells (Figure 9H), a finding highly consistent with our observations in the UUO model.

In summary, this study demonstrates that pharmacological inhibition of FGFR1 effectively attenuates renal pathology, preserves kidney function, reduces extracellular matrix accumulation, alleviates local inflammation, and improves vascular barrier integrity in the UUO model of renal fibrosis. The concurrent activation of the FGFR1/YAP1 axis in the DKD model further underscores the potential clinical relevance of this pathway across different kidney diseases. Combined with our previous genetic evidence from endothelial-specific YAP1 knockout mice, this work provides a robust preclinical foundation for targeting the FGFR1/YAP1 signaling axis as a potential anti-fibrotic therapeutic strategy.

## 4 Discussion

Renal fibrosis represents a common terminal pathway in the progression of all CKD to ESRD. While current therapeutic strategies primarily targeting underlying causes, such as hypertension and hyperglycemia, can partially delay CKD progression (Kishi et al., 2024), there remains a critical lack of treatments that directly target and reverse the fibrotic process itself. Consequently, the development of specific anti-fibrotic therapies constitutes a major unmet need in nephrology. This study focuses on endothelial cells—key contributors to the renal fibrotic microenvironment—and, for the first time, systematically elucidates the central role of the FGFR1/YAP1 signaling axis, mediated by ERK phosphorylation, in driving renal fibrosis through these cells. We demonstrated the coordinated upregulation of FGFR1 and YAP1 in ECs within human fibrotic kidney specimens, a UUO model, and a DKD mouse model. Mechanistically, we established that TGF-β induces EndMT via activation of the FGFR1-ERK-YAP1 axis, thereby promoting inflammatory responses and myofibroblast activation. Importantly, endothelial-specific YAP1 knockout or pharmacological inhibition of its upstream regulator FGFR1 effectively blocks ERK phosphorylation and subsequent YAP1 activation, thereby attenuating renal fibrosis and improving renal function. These findings not only uncover a novel pathogenic mechanism but, more significantly, propose a promising anti-fibrotic strategy: precision intervention via targeting the druggable receptor FGFR1 to circumvent the challenges associated with directly inhibiting the ‘undruggable’ transcriptional coactivator YAP1.

Research on renal fibrosis has traditionally centered on renal tubular epithelial cells and myofibroblasts. However, the focal distribution pattern observed in pathological sections prompted us to consider the role of the local microenvironment: whether specific microenvironment are more “permissive” for fibrosis initiation and propagation. The “vascular niche” theory proposed by Chen et al. (2021) posits that vascular cells actively regulate the function and phenotype of surrounding parenchymal cells (such as tubular epithelial cells and fibroblasts) through paracrine signaling, thereby playing an orchestrating role in fibrosis. Building upon this theoretical framework, we directed our attention to endothelial cells. Our results demonstrate that TGF-β-induced endothelial injury and EndMT are accompanied by profound alterations in the endothelial paracrine profile, a process dependent on the FGFR1-ERK-YAP1 signaling cascade. Notably, inhibition of FGFR1 not only abrogated EndMT by blocking ERK phosphorylation and subsequent YAP1 upregulation but also reshaped the vascular niche: it significantly downregulated endothelial expression of the immune cell adhesion molecule VCAM1 and the pro-inflammatory cytokines IL-6 and TNF-α—thereby limiting immune cell recruitment—while also reducing the secretion of CCN2, a paracrine factor known to activate fibroblasts, consequently attenuating fibroblast activation signals. This notion is supported by Abedini et al., who reported that activated endothelial cells serve as key initiators of local immune responses within the fibrotic niche, recruiting immune cells via chemokines such as CXCL12 to drive disease progression (Abedini et al., 2024). Furthermore, as the frontline interface with systemic circulation, renal ECs are primary sensors of risk factors such as hypertension and hyperglycemia (Lan et al., 2024; Chu et al., 2024; Yang and Liu, 2022). Our data suggest that these stress signals may converge on the FGFR1-ERK-YAP1 axis, triggering pro-fibrotic endothelial activation. Accordingly, we propose that endothelial cells constitute the local microenvironment (“soil”) that determines the fate of parenchymal cells (“seeds”), serving as a critical hub integrating systemic injury signals with local fibrotic programming through the FGFR1-ERK-YAP1 cascade. In fibrotic kidney tissues from patients with advanced CKD, we observed significant, concurrent upregulation of FGFR1 and YAP1 specifically in ECs, suggesting activation of this axis may be a common event in CKD progression across various etiologies. This observation introduces the well-characterized FGFR1-Hippo/YAP1 signaling nexus, prominent in development and oncology, into the realm of renal fibrosis research (Pichol-Thievend et al., 2024). Subsequent *in vitro* and *in vivo* experiments further confirmed that intervention targeting the endothelial FGFR1-ERK-YAP1 axis effectively regulates the fibrotic process, providing robust experimental evidence for endothelial cell-targeted therapy in renal fibrosis—a notion that aligns with findings from Pohl et al. (Pohl and Schiessl, 2023) emphasizing the importance of endothelial cells.

The involvement of the Hippo/YAP1 pathway in renal fibrosis is well-established (Wang et al., 2024). Nonetheless, clinically viable drugs directly targeting this pathway are lacking (Nguyen and Yi, 2019). The commonly used experimental inhibitor verteporfin suffers from non-specificity, requiring high concentrations for efficacy (Nguyen and Yi, 2019; Yong et al., 2021), and has been reported to potentially exacerbate podocyte injury in diabetic nephropathy models (Qi et al., 2022; Zhuang et al., 2021), underscoring the need for alternative intervention strategies. Our study identifies FGFR1 as a key upstream regulator of YAP1 in this context, and further delineates ERK as a critical downstream mediator of FGFR1-induced YAP1 activation: FGFR1 knockdown effectively suppressed ERK phosphorylation and subsequent YAP1 expression, consistent with reports by Pichol-Thievend et al. (2024), Azad et al. (2020) that link FGFR1 to Hippo/YAP1 signaling, and extending these findings to define a clear FGFR1-ERK-YAP1 molecular cascade in renal fibrosisThis positions FGFR1 as a feasible indirect target for YAP1 pathway modulation, with ERK serving as a key actionable node within this axis—one that could also represent a potential secondary therapeutic target, though FGFR1 remains the more attractive candidate given its cell membrane localization and well-characterized inhibitor repertoire.

Notably, our finding that FGFR1 inhibition alleviates fibrosis in the UUO model appears to contrast with studies proposing a protective role for FGFR1. We propose this discrepancy highlights the “context-dependent” nature of FGFR1 signaling. In models like STZ-induced diabetic nephropathy, characterized by absolute insulin deficiency and sustained hyperglycemia, FGFR1 signaling may contribute to endothelial homeostasis (Gao et al., 2023; Li et al., 2020). Conversely, in the UUO model and under high-intensity TGF-β stimulation—contexts dominated by robust inflammation and mechanical stress—FGFR1 signaling is functionally “reprogrammed” to cooperate with YAP1, driving a pro-fibrotic EndMT program. Such context-dependent functional switching of signaling molecules is not uncommon. For instance, Cheng et al. found FGFR1 suppression in high-glucose-treated renal tubular cells, and its restoration was protective (Cheng et al., 2014), whereas Životić et al. reported that FGFR inhibitors effectively inhibited EMT in human renal tubular cells (Životić et al., 2018). To further validate our findings, we conducted experiments in a more clinically relevant diabetic kidney disease mouse model (db/db mice). The results showed that 24-week-old db/db mice developed significant renal interstitial fibrosis, with findings highly consistent with those observed in the UUO model: enhanced FGFR1 and YAP1 co-expression in endothelial cells, accompanied by elevated ERK phosphorylation and increased renal fibrosis. The present findings demonstrate that the endothelial FGFR1/YAP1 signaling axis plays a critical role in the pathogenesis of renal fibrosis, thereby emphasizing that the precise tailoring of future targeted therapies must be guided by specific disease etiology, stage, and pathological microenvironment.

A key translational contribution of this study is a strategic pivot: from directly targeting the ‘undruggable’ nuclear coactivator YAP1 to intervening via its upstream, more tractable membrane receptor, FGFR1—with the FGFR1-ERK-YAP1 axis defined as the critical molecular pathway linking these two nodes. We first established “proof of concept” through endothelial-specific YAP1 knockout, confirming the axis’s significance and the efficacy of its inhibition while avoiding risks associated with systemic YAP1 ablation, thereby providing crucial genetic evidence for precision targeting. Building on this, pharmacological FGFR1 inhibition in the UUO model blocked ERK phosphorylation and YAP1 upregulation, improved renal function and reduced fibrosis, completing the “feasibility verification.” Given that FGFR1 is a receptor tyrosine kinase with a well-developed repertoire of inhibitors (Katoh et al., 2024), our findings provide a strong rationale for expediting the preclinical evaluation of FGFR1-targeted agents for renal indications.

This study has several limitations. For example, we have not yet achieved precision delivery of therapeutic agents specifically to renal endothelial cells. Additionally, the mechanistic validation was primarily conducted in the UUO model and murine DKD models; although human clinical samples were used for correlative analysis, direct causal validation in the human renal fibrosis microenvironment remains lacking. The issues mentioned above represent key directions that our future research needs to focus on and address. In conclusion, this study proposes and validates a viable alternative strategy for intervening against the core fibrotic driver YAP1: precise targeting of its upstream, druggable receptor FGFR1 with the FGFR1-ERK-YAP1 signaling cascade defined as the critical molecular mechanism mediating endothelial-driven renal fibrosis. We have not only revealed the central role of the endothelial FGFR1-ERK-YAP1 axis in driving fibrosis in the UUO model but also demonstrated the effectiveness and feasibility of this approach through complementary genetic and pharmacological proofs-of-concept, with validation in a clinically relevant DKD model. This work provides valuable experimental evidence for developing targeted anti-fibrotic strategies against renal fibrosis, identifies FGFR1 as a potential preclinical target, and highlights the FGFR1-ERK-YAP1 cascade as a key molecular pathway for future mechanistic and therapeutic exploration.

## Data Availability

The original contributions presented in the study are included in the article/Supplementary Material, further inquiries can be directed to the corresponding authors.
