# Supplementary material for: FGFR1/YAP1 signaling in endothelial cells drives renal fibrosis and offers a therapeutic target
Source: Front Pharmacol. 2026 Apr 13;17:1766265. doi: 10.3389/fphar.2026.1766265 (PMC13111247; doi:10.3389/fphar.2026.1766265)
Supplement: Supplementary file 1 [file Supplementaryfile1.docx]

**Supplemental materials**

**Materials and methods**

**Sequences using for shRNA, plasmids constructions, and the real-time PCR**

**Table S1. List of the primers used for the plasmids construction**

| **Genes** | **Forward (5’→3’)** | **Reverse (5’→3’)** |
| --- | --- | --- |
| **FGFR1** | **CCGG** CGAGGCATTATTTGACCGGAT **CTCGAG** ATCCGGTCAAATAATGCCTCG **TTTTT G** | **AATTCAAAAA** CGAGGCATTATTTGACCGGAT **CTCGAG** ATCCGGTCAAATAATGCCTCG **CCGG** |
| **YAP1** | **CCGG** GCCACCAAGCTAGATAAAGAA **CTCGAG** TTCTTTATCTAGCTTGGTGGC **TTTTT G** | **AATTCAAAAA** GCCACCAAGCTAGATAAAGAA **CTCGAG** TTCTTTATCTAGCTTGGTGGC **CCGG** |

**Table S2. Sequences of the indicated shRNA**

| **Target** | **Sequence** |
| --- | --- |
| **shFGFR1** | 5’-CGAGGCATTATTTGACCGGAT-3’ |
| **shYAP1** | 5’-GCCACCAAGCTAGATAAAGAA-3’ |
|  | **Core Sequence** |
| **shNC** | 5’-CCTAAGGTTAAGTCGCCCTCGCTCGAGCGAGGGCGACTTAACCTTAGG-3’ |

**Table S3. List of the primers used for the real-time PCR**

| **Species** | **Genes** | **Forward (5’→3’)** | **Reverse (5’→3’)** |
| --- | --- | --- | --- |
| **Human** | **FGFR1** | AACCTGACCACAGAATTGGAGGCT | ATGCTGCCGTACTCATTCTCCACA |
|  | **YAP1** | CGCTCTTCAACGCCGTCA | AGTACTGGCCTGTCGGGAGT |
|  | **ACTA2** | ACTGCCTTGGTGTGTGACAA | CACCATCACCCCCTGATGTC |
|  | **Vimentin** | GCTTCAGAGAGAGGAAGCCGAAAA | CCGTGAGGTCAGGCTTGGAAA |
|  | **VCAM1** | CATCCACAAAGCTGCAAGAAG | AGGTGCTGTAGATTCCCATTATC |
|  | **IL-6** | CACTGGTCTTTTGGAGTTTGAG | GGACTTTTGTACTCATCTGCAC |
|  | **TNF-α** | CAGAGGGAAGAGTTCCCCAG | CCTTGGTCTGGTAGGAGACG |
|  | **Ang2** | AACTTTCGGAAGAGCATGGAC | CGAGTCATCGTATTCGAGCGG |
|  | **CCN2** | TTAAGAAGGGCAAAAAGTGC | CATACTCCACAGAATTTAGCTC |
|  | **GAPDH** | TATGACAACAGCCTCAAGAT | AGTCCTTCCACGATACCA |
|  | **β-actin** | GGACTTCGAGCAAGAGATGG | AGCACTGTGTTGGCGTACAG |
| **Mouse** | **YAP1** | ACCCTCGTTTTGCCATGAAC | TTGTTTCAACCGCAGTCTCTC |
|  | **ACTA2** | CCCAACTGGGACCACATGG | TACATGCGGGGGACATTGAAG |
|  | **Fibronectin** | GAGCTATCCATTTCACCTTCAGA | TTGTTCGTAGACACTGGAGAC |
|  | **CollagenⅠ** | CGCAAAGAGTCTACATGTCTAGG | CATTGTGTATGCAGCTGACTTC |
|  | **VCAM1** | CTTGTGGAAATGTGCCCGAAAC | TGTGCCTGGCGGATGGTGTA |
|  | **IL-6** | CTCTGGGAAATCGTGGAAATG | AAGTGCATCATGGTTGTTCAT |
|  | **TNF-α** | ATTCGAGTGACAAGCCTGTAG | TGAAGAGAACCTGGGAGT |
|  | **Ang2** | CCTCTGGGAGAGTACTGGCT | GCTACTTATTTTGCCCGCGG |
|  | **CCN2** | GCTGACCTGGAGGAAAACATTAA | TGACAGGCTTGGCGATTTTAG |
|  | **GAPDH** | AAGGTCATCCCAGAGCTGAA | CTGCTTCACCACCTTCTTGA |

**Supplement Figure 1**

**
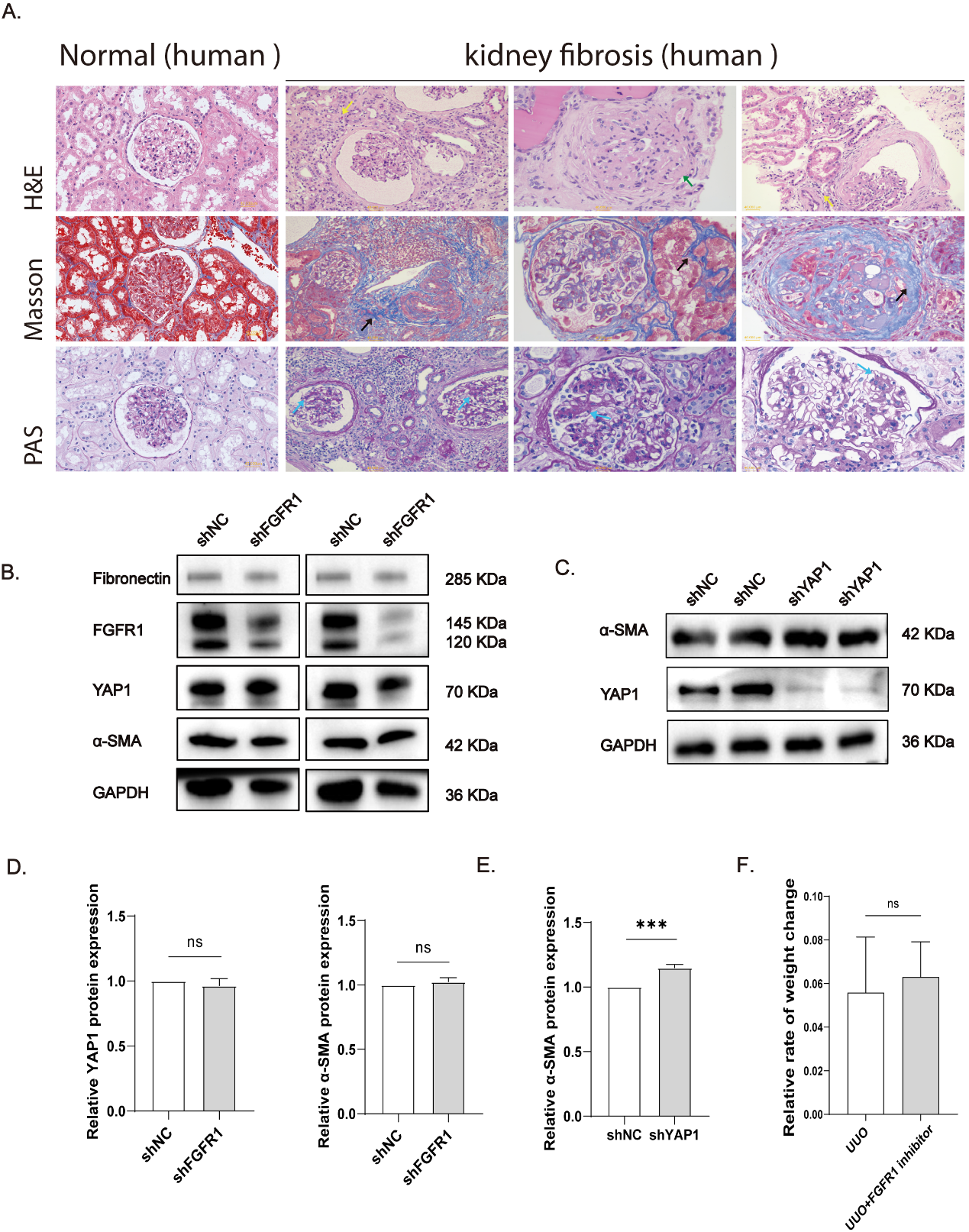
**

**Fig.S1.** **Histopathological features of renal fibrosis and the regulation of fibrotic markers by YAP1 and FGFR1 knockdown.** (A) Representative HE, Masson's trichrome, and PAS staining images showing renal fibrosis in human renal tissues. HE staining reveals inflammatory cell infiltration (yellow arrows) and glomerular ischemic collapse with periglomerular fibrosis and hyalinosis (green arrow). Masson's trichrome staining highlights blue collagen fiber deposition (black arrow), and PAS staining demonstrates mesangial matrix and GBM expansion (blue arrows). Bar=40μm. (B and D) Western blot analysis revealed that the protein expression levels of YAP1, α-SMA, and fibronectin in the shFGFR1 group were comparable to those in the shNC control group, indicating that FGFR1 knockdown did not significantly modulate the baseline expression of these markers. n=3. (C) Western blot analysis revealed that knockdown of YAP1 resulted in a modest increase in α-SMA expression; (E) semi-quantitative analysis showed that the increase in α-SMA was statistically significant. n=3. (F) Analysis of the relative body weight change rate on day 10 post-administration showed that the body weight loss trend induced by UUO surgery was not further exacerbated by PD173074 treatment. There was no statistically significant difference in body weight change between the treatment group and the UUO group, indicating that the drug intervention did not significantly affect the general systemic condition (assessed by body weight) of the mice at this time point. n=5.***p<0.001;ns, no significance.

**Supplement Figure 2**

**
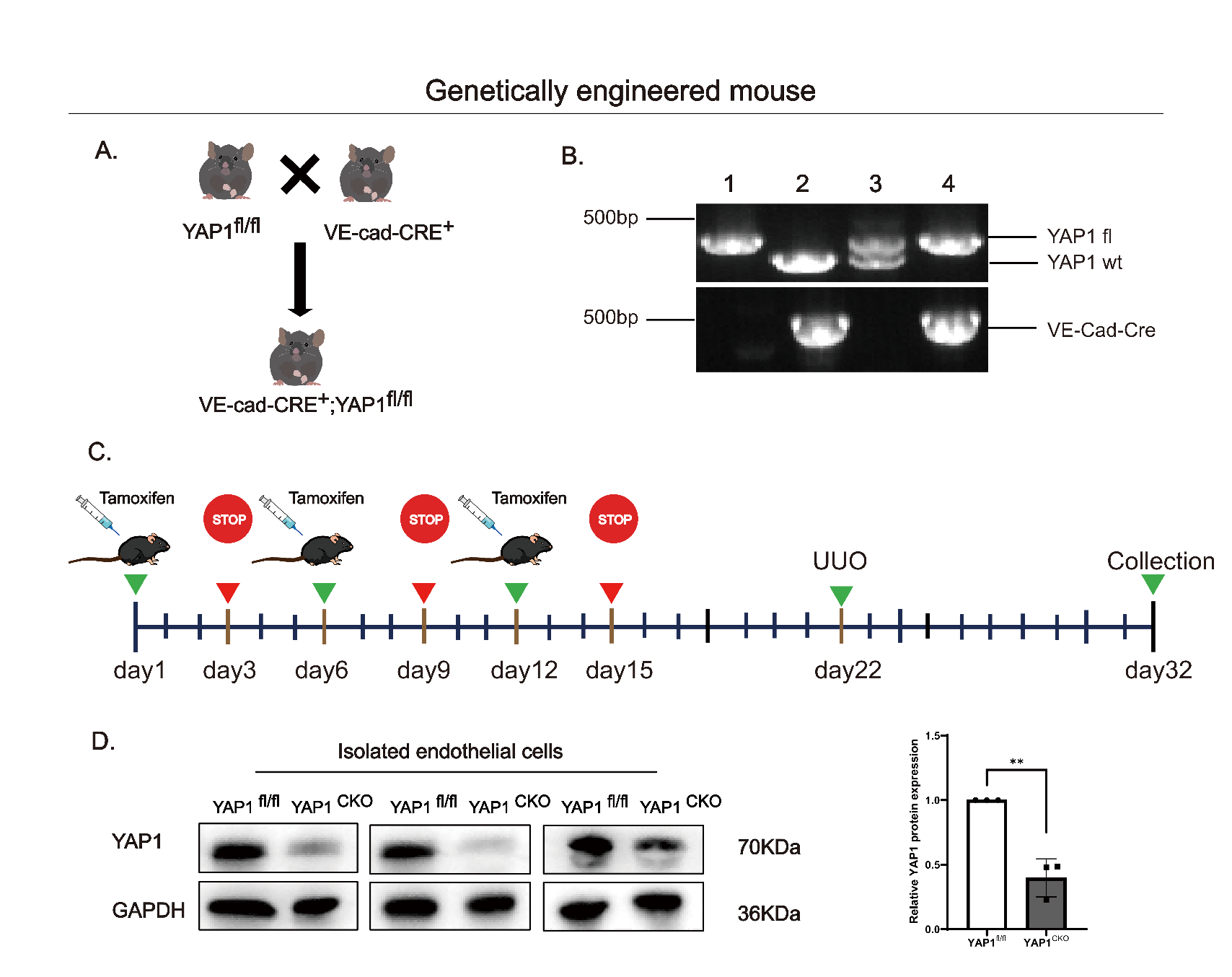
**

**Fig.S2.Generation of YAP1^fl/fl^VE-Cad-Cre^+/-^(YAP^CKO^) mice and genotype**(A) Schematic of the breeding strategy for generating YAP1^fl/fl^VE-Cad-Cre^+/-^(YAP^CKO^) mice.(B) Representative gel electrophoresis of PCR genotyping.Lane1: YAP1^fl/fl^VE-Cad-Cre^-/-^;Lane2:YAP1^wt/wt^VE-Cad-Cre^+/-^;Lane3:YAP1^fl/wt^VE-Cad-Cre^-/-^;Lane4:YAP1^fl/fl^VE-Cad-Cre^+/-^.(C) Schematic timeline of tamoxifen-induced endothelial-specific YAP1 knockout, subsequent model establishment, and tissue collection in mice. (D) Left, Western blot analysis confirmed the efficiency of YAP1 knockout: YAP1 protein expression was markedly lower in the kidneys of YAP1^CKO^ mice compared with the YAP1^fl/fl^ control group. Right: Quantification of relative YAP protein expression (densitometry); YAP1^fl/fl^ group set as 1 arbitrary unit n=3.Data are presented as mean + SD. **p<0.01.
